# Urinary Sodium and Potassium Excretion in Bangladeshi Adults: Results from a Population-Based Survey with 24-Hour Urine Collections

**DOI:** 10.5334/gh.1447

**Published:** 2025-07-14

**Authors:** Jubaida Akhtar, Mohammad A. Al-Mamun, Mohammad N.-N. Sayem, Mohammad J. Ahmed, Mahfuzur R. Bhuiyan, Shamim Jubayer, Mohammad R. Amin, R. Karim, Megan E. Henry, Matti Marklund, Laura Cobb, Dinesh Neupane, Lawrence J. Appel, Sohel R. Choudhury

**Affiliations:** 1 Department of Epidemiology and Research, National Heart Foundation Hospital and Research Institute, Mirpur-1, Dhaka, Bangladesh; 2 Department of Pathology, National Heart Foundation Hospital and Research Institute, Mirpur-1, Dhaka, Bangladesh; 3 Non-Communicable Disease Control (NCDC) Programme, Directorate General of Health Services (DGHS), Mohakhali, Dhaka, Bangladesh; 4 Department of Epidemiology, Bloomberg School of Public Health, Johns Hopkins University, Baltimore, Maryland, USA; 5 Resolve To Save Lives (RTSL), Vital Strategies, New York, USA; 6 Department of International Health, Bloomberg School of Public Health, Johns Hopkins University, US; 7 Welch Center for Prevention, Epidemiology, and Clinical Research, Johns Hopkins University, Baltimore, Maryland, USA

**Keywords:** Sodium, Potassium, Dietary sodium, Dietary salt, Dietary potassium, Bangladesh

## Abstract

**Introduction:**

The high burden of blood pressure-related cardiovascular diseases in Bangladesh is potentially caused by excessive dietary sodium and insufficient potassium intake. Our objective is to estimate dietary salt and potassium intake among Bangladesh rural and urban adults from urinary excretion of sodium and potassium.

**Methods:**

We conducted a cross-sectional study between December 2017 and June 2018, including participants aged 30–59 years from three urban and three rural sites in Bangladesh. Data included urinary excretion of sodium and potassium estimated from one 24-hr urine collection and blood pressure measurements.

**Results:**

Among 840 enrolled participants, complete data was available in 509 individuals. Mean age was 43.0 (SD ±7.9) years; 20.9% had hypertension, 50.9% were women, and 50.9% resided in urban areas. Mean systolic and diastolic blood pressure were 118.6 (SD ± 16.6) mmHg and 76.3 (SD ± 11.3) mmHg, respectively. Overall, the mean urinary sodium excretion was 3.9 g/day (95% CI = 3.8 to 4.0), corresponding to a mean salt intake of 9.7 g/day (95% CI = 9.4–10.1). Mean urinary potassium excretion was 1.4 g/day (95% CI = 1.3–1.4), corresponding to an estimated mean dietary potassium intake of 2.0 g/day. Men and urban residents had slightly but non-significantly higher sodium and potassium excretion than women and rural residents.

**Conclusion:**

In Bangladesh, salt intake exceeded WHO’s recommended <5g/day limit, while potassium intake was substantially lower than the recommended intake of ≥ 3.5g/day for adults. Promoting low-sodium and potassium-rich diets through nationwide campaigns and policies, including advocating for accessible low-sodium and potassium-enriched salt substitutes, is recommended to mitigate cardiovascular disease risks.

## Introduction

Hypertension is a preventable risk factor for non-communicable diseases (NCD) (1) and a significant contributor to cardiovascular morbidity and disability (2). The Global Burden of Disease Study estimates that hypertension is responsible for one-third of global deaths annually (3). According to the 2022 Bangladesh NCD Risk Factor Survey, approximately 24.6% of adults have hypertension (4). Concurrently, there is a substantial burden of stroke in Bangladesh, with a prevalence of 11.39 per 1000 adults (5). Additionally, NCDs are responsible for 67% of all deaths, and approximately 30% of total fatalities are attributed to cardiovascular diseases (CVD) in Bangladesh (6).

Dietary lifestyle factors have a major role in the development of hypertension (7). High dietary salt (sodium chloride) intake is one of the most important dietary factors that raise blood pressure (BP) and increase the risk of hypertension (8,9). In 2019, over 40.5 million disability-adjusted life years were lost due to high sodium intake-related CVD (2). Population-wide salt reduction has been recommended as a key strategy for preventing and controlling hypertension (10). Prevention of hypertension by reducing salt intake is deemed one of the most cost-effective approaches for CVD prevention and control (11,12). The World Health Organization (WHO) recommended dietary intake of salt is <5 g/day (<2 g/day sodium) for adults (12). However, global salt intake varies from 9 to 12 g/day, greatly exceeding the WHO recommendation (12). A large body of evidence, including over 100 clinical trials, has documented that as salt intake rises, BP increases (13,14). Likewise, a low dietary potassium intake raises BP and increases the risk of developing hypertension and its complications (9,15). A low potassium intake also increases the pressor effects of sodium (dietary salt) on BP (15). The WHO recommends a daily potassium intake of at least 3.5 g (90 mmol or 3510 mg) to lower BP (15).

Considering the adverse health effects of excess sodium and insufficient potassium intake, it is crucial to estimate the average intake of these dietary factors at a population level. Such information can serve as a baseline and guide public health initiatives to prevent and control hypertension (16). Of the available dietary assessment tools, urinary sodium and potassium excretion is the most accurate method to estimate their intake (15,17). Usually, under normal conditions, a small amount of sodium is lost through sweat or feces, and almost all of the sodium consumed through diet is excreted in the urine over 24-hr period (18). In contrast, 60–80% of dietary potassium is excreted in the urine over the same period (19). In Bangladesh, only a few studies have used 24-hr urine collection to assess salt intake, but none have reported urinary potassium excretion. In this study, we measured urinary sodium and potassium excretion to estimate both dietary salt and potassium intake among Bangladeshi adults.

## Methods

Between December 2017 and June 2018, a cross-sectional study was conducted among community residents of three purposively selected divisions of Bangladesh. Then we selected two clusters (one urban site and one rural site) from each division. We selected Bagerhat municipality town as urban and Paturpara village of Bagerhat district as rural sites from Khulna division; Kafrul ward no.16 of Dhaka North City Corporation as urban, and Taljanga union of Tarail sub-district as rural sites from Dhaka division; and Jaldhaka sub-district of Nilphamari district as rural and Rangpur city, ward no. 01 of Rangpur City Corporation as urban from Rangpur division. Dhaka is located in the country’s south-central region, Rangpur is in the northern region, and Khulna is in the southwest.

Community-dwelling men and women aged 30 to 59 were eligible for the study. Those with a known history of heart failure or kidney disease, stroke, liver failure, terminal illness (cancer), use of diuretics, or those altering their dietary practices were excluded. Pregnant women were also excluded.

Initially, six study clinics were set up at the study sites to facilitate the participants. Before collecting data, the researchers recruited study staff and provided them with an overview of the study, followed by comprehensive training. Upon completing training, the research staff compiled a list of 300 households within the catchment areas, specifically focusing on households where individuals aged 30 to 59 had been residing for at least six months. Invitation letters were sent to these individuals by the study staff, inviting them to participate in the study. Participants were invited to study clinics set up at the sites for enrollment, following a “first-come, first-enrolled” approach until 140 enrolled. Only one eligible participant was selected from each household. Enrollment at each site was stratified by age (30–44 and 45–59) and gender to ensure that 25% of participants represented men aged 30–44, men aged 45–59, women aged 30–44, and women aged 45–59.

Participants’ demographic and clinical history information was collected using a modified WHO NCD risk factor surveillance (STEPS) survey questionnaire (20). The measurements included BP and anthropometry (height and weight). The study physician measured BP using an electronic sphygmomanometer (Omron, Model No: JPN1 (HEM-7200-AP3)). Participants were comfortably seated for 5 minutes before BP measurements were taken on the right arm. Two separate measurements were obtained, and the average of these two measurements was recorded. Persons were considered hypertensive if the average of two systolic blood pressure (SBP) measurements was ≥140 mmHg and/or diastolic blood pressure (DBP) ≥90 mmHg or they self-reported the use of antihypertensive medication.

Participants’ height was measured with a stadiometer, and weight was recorded using a digital weighing machine (Model No: BC-541N TANITA). Body Mass Index was calculated by dividing the body weight in kilograms by the square of the height in meters.

### 24-hr urine collection and analysis

All participants received written and verbal instructions on collecting the 24-hr urine sample. They were instructed to discard the first-morning urine at the start of the day and then collect subsequent urine for the next 24 hrs, recording the exact start and finish time of the collection. Urine collection containers were returned, and the total volume of the collection was measured. A 20 ml aliquot of urine samples was extracted, stored at –20°C, and sent to the laboratory for analysis. All tests were done at the biochemical laboratory of the National Heart Foundation Hospital and Research Institute by using an auto-analyzer (Easylyte, Medica). The sodium and potassium excretion over 24-hr, and dietary salt and potassium intake per day were estimated following the WHO protocol (21). The urine samples were excluded if the total urine volume was very low (<0.5 liter/day) or very high (>4.5 liter/day) or if the total creatinine excretion, corrected for body weight, was outside the range of 14.4–33.6 mg/kg for men and 10.8–25.2 mg/kg for women (22).

Estimates of urinary sodium and potassium excretion were reported in g/day. The 24-hr sodium excretion was calculated by multiplying the sodium concentration (mmol/L) by the urine volume (L) and then converting it to mg/day by multiplying by 23 (the molar mass of sodium is 23 g/mol). To estimate salt intake (sodium chloride), we multiplied urinary sodium excretion by 2.5 (1 mg sodium = 2.5 mg salt) and divided the resulting value by 1000 (1 g = 1000 mg) to obtain salt intake in g/day. The 24-hr potassium excretion was calculated by multiplying the potassium concentration (mmol/L) by the urine volume (L) and then converted to mg/day by multiplying by 39.1 (the molar mass of potassium is 39.1 g/mol). The resulting value was divided by 1000 (1 g = 1000 mg) to express urinary potassium excretion in g/day. To estimate average daily potassium intake, we divided urinary potassium excretion by 0.7 (19).

The sample size was determined following the recommended WHO protocol (23). To detect approximately a 20 mmol reduction in sodium intake over time using 24-hr urinary sodium excretion, with a standard deviation of 60 mmol/day (alpha = 0.05, power = 0.80), a minimum sample of 119 individuals per age and sex stratum is recommended. In this study, we determined the sample size using the formula: n = 2σ²/Δ² (Zα + Zβ)², where Za = 1.96 for a = 0.05 (two-tailed) and Zb = 0.84 for 80% power. Therefore, 140 eligible participants were enrolled from each site, and the total sample size was 840 (140 × 6) from six study clinics.

National Heart Foundation Hospital and Research Institute Review Committee reviewed and approved this study. [Ref: N.H.F.H. & R.I. 4–14/7/Ad/852 dated 31/03/2018]. All study participants provided written informed consent.

### Statistical analysis

Categorical variables were presented as frequencies and percentages, and continuous variables as mean and standard deviations (SD) for summary measures. Mean and 95% confidence interval (CI) were calculated for all continuous variables. Mean and 95% CI were calculated for urinary sodium and potassium as unadjusted and adjusted by age and sex using linear regression. The statistical software Stata version 17 was used for all analyses, with significance considered at a 95% CI.

## Results

Of 840 participants enrolled in this study, 509 were included in the analysis. We excluded 331 individuals because their 24-hr urine samples did not meet our quality control thresholds; 290 participants were excluded based on creatinine excretions that fell outside the pre-specified range, 30 participants based on urine volume criteria, 10 participants based on extreme outliers of sodium and potassium, and one participant based on incomplete information.

An approximately equal number of men and women were included in the study. Likewise, approximately half (50.9%) came from urban areas. The mean age of the participants was 43.0 (SD ± 7.9) years; the mean BMI was 23.9 (SD ± 4.2) kg/m^2^, the mean SBP and DBP were 118.6 (SD ± 16.6) mmHg and 76.3 (SD ± 11.3) mmHg, respectively. Most participants (55.4%) reported adding salt during their meals. Among the participants, 20.6% had hypertension. All sociodemographic characteristics of the included participants were comparable to those excluded from the analysis. Among the excluded participants, 48% were female, and 48.6% lived in urban areas. The mean age of the excluded participants was 44 (SD: ± 8.3) years, mean BMI was 24.5 (SD ± 4.7), mean SBP and DBP were 121.8 (SD ± 19.0) mmHg and 78.3 (SD ± 12.1) mmHg, respectively. However, the excluded group had a significantly higher prevalence of hypertension (33.3% vs. 20.6%, *p* < 0.001) (Table 1).

**Table 1:** Baseline characteristics of urinary sodium and potassium excretion of the study participants (both excluded and included), Bangladesh, December 2017 and June 2018.


VARIABLES	INCLUDED (N = 509)	EXCLUDED (N = 330)	P VALUE

Age (years)	43.0 ± 7.9	44.0 ± 8.3	0.083

Female	259 (50.9)	159 (48.2)	0.444

Urban	259 (50.9)	161 (48.8)	0.553

BMI (kg/m^2^)	23.9 ± 4.2	24.5 ± 4.7	0.059

Systolic BP (mmHg)	118.6 ± 16.6	121.8 ± 19.0	0.010

Diastolic BP (mmHg)	76.3 ± 11.3	78.3 ± 12.1	0.013

Hypertension, yes	105 (20.6)	110 (33.3)	<0.001

Diabetes, yes	32 (6.3)	24 (7.3)	0.563

Level of education			0.033

No education	58 (11.4)	62 (18.8)	

Primary level	218 (42.8)	119 (36.2)	

Secondary level	142 (27.9)	95 (28.9)	

Higher secondary level	40 (7.9)	22 (6.7)	

Graduation and above	51 (10.0)	31 (9.4)	

Add salt during meal			0.188

Never	227 (44.6)	147 (44.6)	

Rarely	33 (6.5)	36 (10.9)	

Sometimes	136 (26.7)	78 (23.6)	

Often	35 (6.9)	18 (5.5)	

Always	78 (15.3)	51 (15.5)	

Amount of extra salt added during meal (n = 465)			0.509

One pinch	229 (81.2)	153 (83.6)	

More than one pinch	53 (18.8)	30 (16.4)	

Daily smoker	109 (21.4)	67 (20.3)	0.699

Daily smokeless tobacco user	158 (31.0)	102 (30.9)	0.968


N = Number of observations; SD = Standard Deviation; n = Frequency; BP: Blood pressure; BMI: Body Mass Index. **Note:** A meal refers to consuming a substantial amount of food at regular intervals throughout the day, such as breakfast, lunch, or supper.

**Figure 1:**
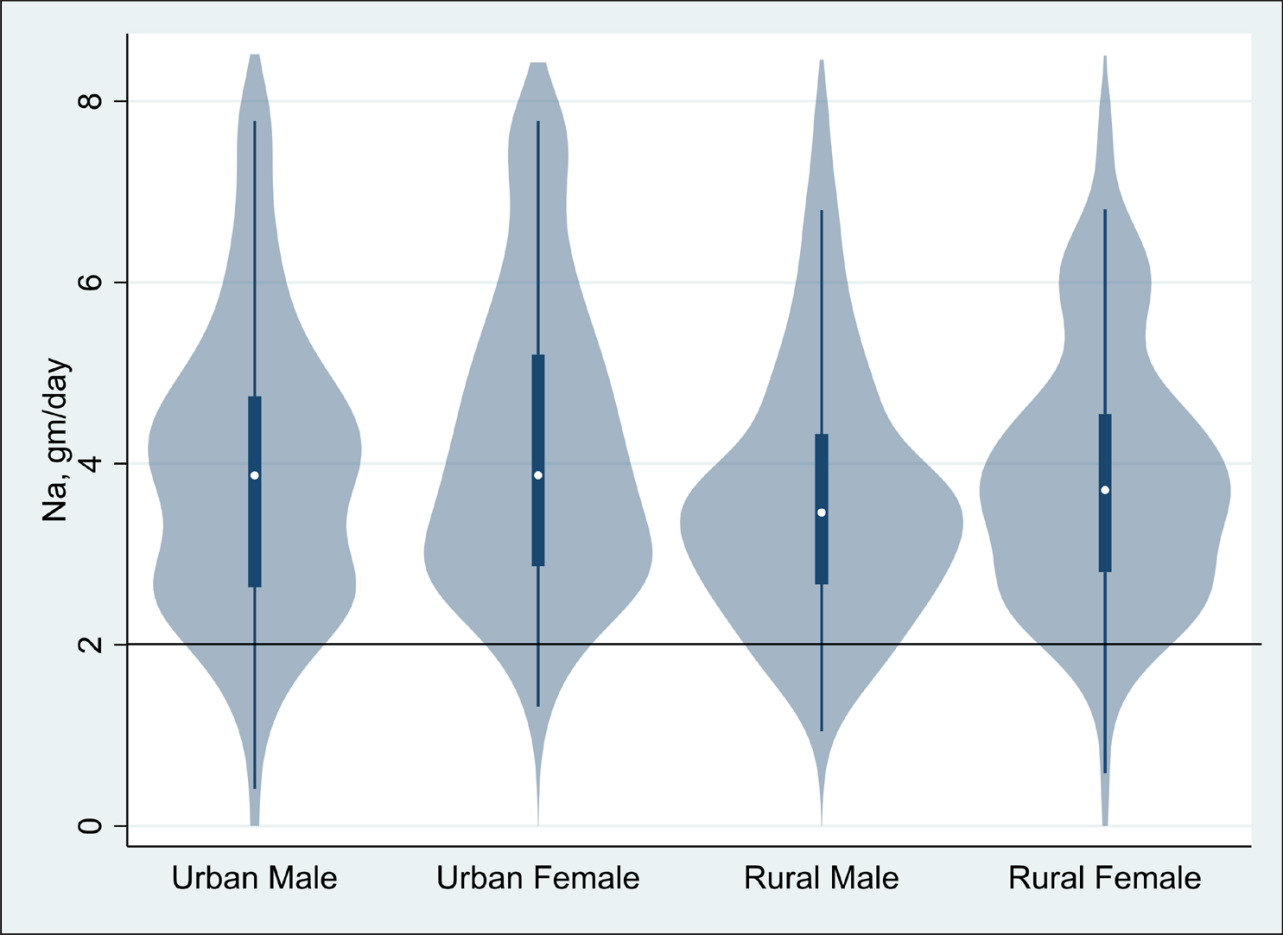
Violin plot of distributions of urinary (dietary) sodium by gender and place of residence of the study participants, Bangladesh, December 2017 and June 2018 (N = 509). The horizontal line at 2 g/d indicates the upper limit of intake recommended by WHO.

**Figure 2:**
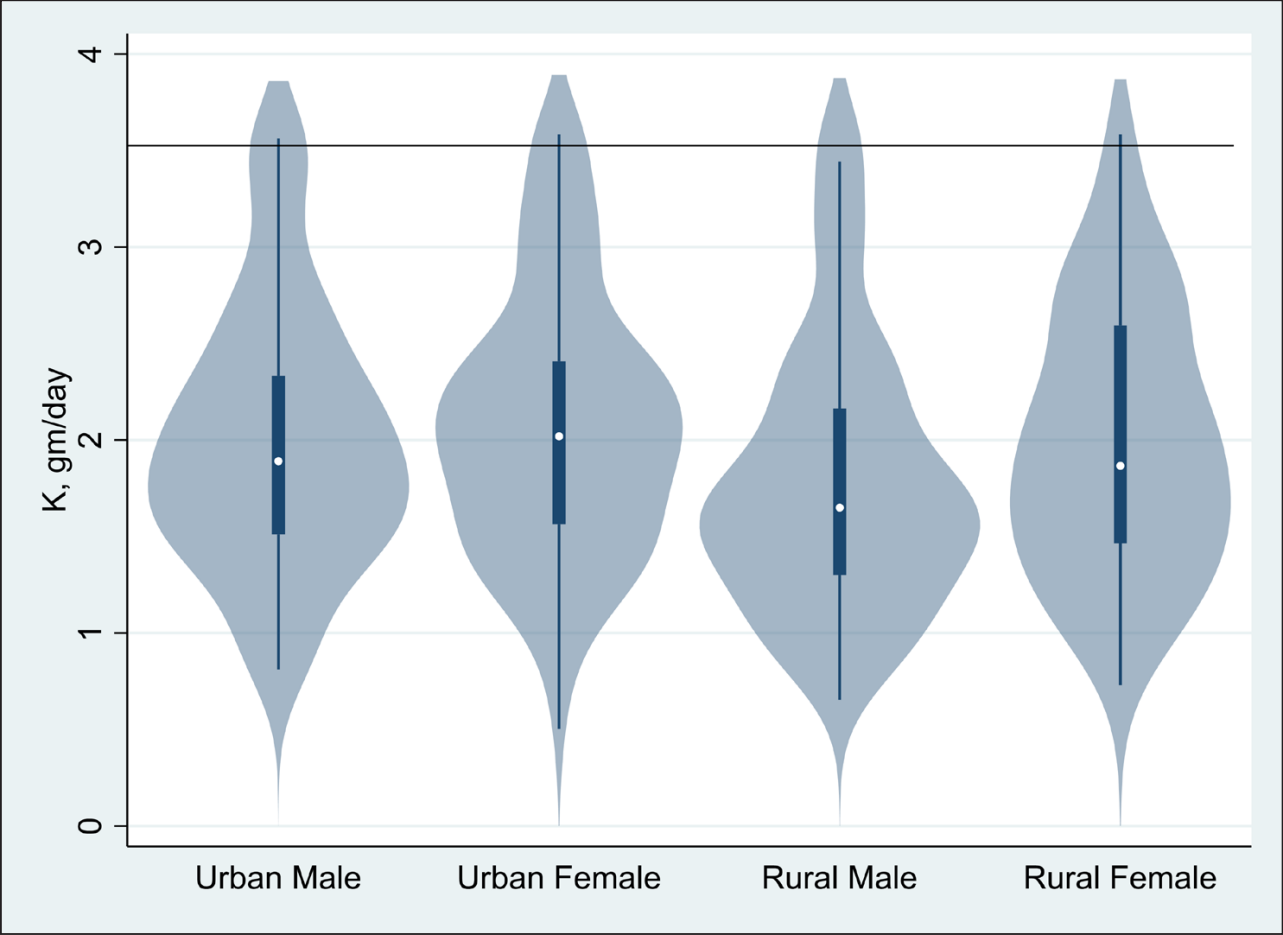
Violin plot of distributions of potassium intake by gender and place of residence of the study participants. The horizontal line at 3.5 g/d indicates the lower limit of intake recommended by WHO.

Mean urinary sodium excretion was 3.9 g/day (95% CI: 3.8–4.0) overall, and 4.0 g/day (95% CI: 3.8–4.2) in men and 3.8 g/day (95% CI: 3.6–4.0) in women. These estimates correspond to the mean estimated salt intake of 9.7 g/day (95% CI = 9.4–10.1) overall and 10.1 g/day (95% CI: 9.6–10.6) in men and 9.4 g/day (95% CI: 9.0–9.9) in women. Mean salt intake was higher among urban participants (10.2 g/day; 95% CI = 9.7–10.6) than rural participants (9.3 g/day; 95% CI = 8.9–9.7). Mean urinary potassium excretion was 1.4 g/day (95% CI: 1.3–1.4) overall, and 1.4 g/day (95% CI: 1.4–1.5) in men and 1.3 g/day (95% CI: 1.3–1.4) in women. These estimates correspond to mean dietary potassium intake of 2.0 g/day (95% CI: 1.9–2.0) overall, and 2.0 g/day (95% CI: 1.9–2.1) in men and 1.9 g/day (95% CI: 1.8–2.0) in women. Mean dietary potassium intake was 2.0 g/day (95% CI: 2.0–2) in urban participants and 1.9 g/day; 95% CI = 1.8–2.0) in rural participants. Figure 1 presents Violin plots illustrating the distribution of measured daily sodium intake; most values exceeded the WHO recommended limit of 2 g/day. Figure 2 shows the distribution of measured daily potassium intake; most values were well below the WHO recommended threshold of 3.5 g/day. Both plots are stratified by gender and place of residence. These plots illustrate the average sodium and potassium intake distribution among participants compared to the recommended WHO intake.

Participants aged 40–49 years consumed more sodium (4.0 g/day; 95% CI = 3.8–4.2) than those in other age groups. Urban residents had a higher sodium consumption (4.1 g/day; 95% CI = 3.9–4.2) than rural residents. Moreover, participants aged 40–49 years consumed more potassium (2.0 g/day; 95% CI = 1.9–2.1) than those in other age groups. Urban residents had a higher potassium intake (2.0 g/day; 95% CI = 2.0–2.1) than rural residents. About 15.3% always added more salt to their meals, while 44.6% did not add. Among the participants, 81.3% believed they were consuming the right amount of salt. Additionally, 40.1% of participants sometimes, rarely, or often added salt to their meals. Half of the participants (51.7%) knew that high salt intake could cause serious health illnesses, while 39.7% did not. Moreover, 50.3% of the participants reported that reducing salt intake in their diet was somewhat important (Table 2).

**Table 2:** Differences in urinary (dietary) sodium, urinary potassium, and dietary potassium by risk factors among the study participants, Bangladesh, December 2017 and June 2018 (N = 509).


VARIABLES	n (%)	URINARY SODIUM (g/DAY)		URINARY POTASSIUM (g/DAY)		DIETARY POTASSIUM (g/DAY)	
		
		MEAN (95% CI)	*ADJUSTED MEAN (95%CI)	MEAN (95% CI)	*ADJUSTED MEAN (95% CI)	MEAN (95% CI)	*ADJUSTED MEAN (95% CI)

Age (year)							

30–39	187 (36.7)	3.8 (3.6–4.0)	3.8 (3.6–4.0)	1.3 (1.3–1.4)	1.4 (1.3–1.4)	1.9 (1.8–2.0)	1.9 (1.8–2.0)

40–49	208 (40.9)	4.0 (3.8–4.2)	4.0 (3.8–4.2)	1.4 (1.4–1.5)	1.4 (1.4–1.5)	2.0 (1.9–2.1)	2.0 (1.9–2.1)

50–59	114 (22.4)	3.8 (3.5–4.1)	3.8 (3.5–4.1)	1.3 (1.2–1.4)	1.3 (1.2–1.4)	1.9 (1.8–2.0)	1.9 (1.8–2.0)

Gender							

Female	259 (50.9)	3.8 (3.6–3.9)	3.8 (3.6–3.9)	1.3 (1.3–1.4)	1.3 (1.3–1.4)	1.9 (1.8–2.0)	1.9 (1.8–2.0)

Male	250 (49.1)	4.0 (3.8–4.2)	4.0 (3.8–4.2)	1.4 (1.4–1.5)	1.4 (1.4–1.5)	2.0 (1.9–2.0)	2.0 (1.9–2.1)

Place of residence							

Urban	259 (50.9)	4.1 (3.9–4.2)	4.0 (3.9–4.2)	1.4 (1.4–1.5)	1.4 (1.4–1.5)	2.0 (2.0–2.1)	2.0 (2.0–2.1)

Rural	250 (49.1)	3.7 (3.5–3.9)	3.7 (3.5–3.9)	1.3 (1.3–1.4)	1.3 (1.3–1.4)	1.9 (1.8–2.0)	1.9 (1.8–2.0)

Add salt before eating							

Never	227 (44.6)	3.8 (3.6–4.0)	3.8 (3.6–4.0)	1.4 (1.4–1.5)	1.4 (1.3–1.4)	2.0 (1.9–2.1)	2.0 (1.9–2.1)

Rarely/Sometimes/Often	204 (40.1)	3.9 (3.7–4.1)	3.9 (3.7–4.1)	1.4 (1.3–1.5)	1.4 (1.4–1.5)	2.0 (1.9–2.1)	2.0 (1.9–2.1)

Always	78 (15.3)	4.1 (3.7–4.5)	4.1 (3.8–4.4)	1.3 (1.1–1.4)	1.3 (1.1–1.4)	1.8 (1.6–1.9)	1.8 (1.6–1.9)

How much salt do you think you consume?							

Right amount	414 (81.3)	3.9 (3.7–4.0)	3.9 (3.7–4.0)	1.4 (1.3–1.4)	1.4 (1.3–1.4)	2.0 (1.9–2.0)	2.0 (1.9–2.0)

Low	45 (8.8)	3.8 (3.4–4.2)	3.8 (3.3–4.2)	1.4 (1.3–1.5)	1.4 (1.2–1.5)	2.0 (1.8–2.2)	2.0 (1.8–2.2)

High	50 (9.8)	4.2 (3.6–4.7)	4.2 (3.8–4.6)	1.4 (1.3–1.6)	1.4 (1.3–1.6)	2.0 (1.8–2.2)	2.0 (1.8–2.2)

High salt diet causes a serious health problem							

Yes	263 (51.7)	4.0 (3.8–4.2)	4.0 (3.8–4.2)	1.4 (1.4–1.5)	1.4 (1.4–1.5)	2.0 (2.0–2.1)	2.0 (2.0–2.1)

No	44 (8.6)	3.8 (3.4–4.3)	3.8 (3.4–4.3)	1.4 (1.3–1.6)	1.4 (1.3–1.5)	2.0 (1.8–2.2)	2.0 (1.8–2.2)

Don’t know	202 (39.7)	3.8 (3.6–4.0)	3.8 (3.6–4.0)	1.3 (1.2–1.4)	1.3 (1.2–1.4)	1.8 (1.8–1.9)	1.8 (1.8–1.9)

Importance of salt reduction in food							

Very important	203 (39.9)	4.0 (3.8–4.2)	4.0 (3.8–4.2)	1.4 (1.4–1.5)	1.4 (1.4–1.5)	2.1 (2.0–2.2)	2.1 (2.0–2.2)

Somehow important	256 (50.3)	3.9 (3.7–4.0)	3.9 (3.7–4.0)	1.3 (1.3–1.4)	1.3 (1.3–1.4)	1.9 (1.8–2.0)	1.9 (1.8–2.0)

Not important/Do not know	50 (9.8)	3.7 (3.2–4.1)	3.7 (3.2–4.1)	1.4 (1.2–1.5)	1.4 (1.3–1.5)	2.0 (1.8–2.2)	2.0 (1.8–2.2)


*Gender-adjusted for age, age-adjusted for gender, age and gender-adjusted for other variables; n = Frequency; CI = Confidence Interval.

Table 3 presents multivariable regression results examining the associations of urinary sodium (Na), potassium (K), and the sodium-to-potassium (Na/K) ratio with systolic and diastolic blood pressure (BP), adjusted for age and sex. Among these, only urinary sodium showed a significant positive association with diastolic BP. Table 4 displays logistic regression analyses assessing the relationship of the same urinary biomarkers with the odds of hypertension. After adjusting for age and sex, none of the urinary biomarkers were significantly associated with hypertension.

**Table 3:** Multivariable regression analyses*, associating blood pressure with urinary sodium (Na), potassium (K), and urinary sodium/potassium (Na/K) ratio (n = 509).


	SYSTOLIC BP (mmHg)			DIASTOLIC BP (mmHg)		
	
	β	95% CI OF β	P VALUE	β	95% CI OF β	P VALUE

Na (g/day)	0.91	–0.04–1.85	0.06	0.82	0.17–1.47	0.014

K (g/day)	0.39	–2.53–3.32	0.80	1.32	–0.69–3.33	0.20

Na/K ratio (mmol/L)	0.15	0.54–0.83	0.68	0.12	0.35–0.58	0.63


*Separate analysis for systolic and diastolic BP, and separate for Na, K and Na/K ratio. Each model was adjusted for age and sex.

**Table 4:** Logistic regression* for predicting hypertension with urinary sodium (Na), potassium (K), and urinary sodium/potassium (Na/K) ratio (n = 509).


	ODDS RATIO	95% CI OF ODDS RATIO	P VALUE

Na (gm/day)	1.08	0.94–1.25	0.285

K (gm/day)	1.02	0.74–1.41	0.910

Na/K ratio (mmol/L)	1.05	0.94–1.17	0.410


*Separate analysis for Na, K and Na/K. Each model was adjusted for age and sex.

## Discussion

In this population-based study on Bangladeshi adults living in urban and rural settings, the mean dietary salt intake was 9.7 g/day (95% CI = 9.4–10.1), mean sodium intake was 3.9 g/day (95% CI = 3.8–4.0), and mean potassium intake was 2.0 g/day (95% CI = 1.9–2.0). Men and urban residents had slightly higher sodium and potassium intake than women and rural residents, though the differences were not statistically significant.

These results indicate that mean salt intake among Bangladeshi adults is almost twice the WHO recommended upper limit of 5 g/day (12). This is consistent with previous studies in Bangladesh that reported high salt consumption rates based on 24-hr urine collections (24,25). A systematic review conducted in 2017 revealed a range of mean salt intake in India between 5.22 and 42.30 g/day (26). Mean salt intake was reported as approximately 10 g/day in the South Asian region in 2021 in a review (27). Studies in Nepal, Japan, and China have reported even higher salt intakes (27,28).

Additionally, the study’s estimated mean potassium intake was lower than the WHO-recommended level of at least 3.5 g/day (15). Reddin C. et al. conducted a systematic review in 2023, revealing that the mean potassium intake globally was 2.25 g/day, higher in men (2.40 g/day) than women (2.09 g/day) (29). Furthermore, only 14% (95% CI = 11–17%) of the world’s population met the recommended average potassium consumption (29). Low potassium intake is particularly common in South Asian and Southeast Asian countries; six of the ten countries with the lowest estimated potassium intake are from these two regions (29).

Previous studies have emphasized the effectiveness of interventions, such as a healthy diet, in preventing and managing hypertension (13,30,31). A healthy diet is crucial in managing hypertension, especially in low-income populations (32). In Bangladesh, a lower middle-income country, a significant portion of individuals with high BP remain unaware of their condition (51.3%), with a limited number receiving drug treatment. Only 14.1% of individuals with hypertension have controlled BP while on drug treatment (33). Population-based intervention studies have demonstrated that lowering salt intake reduces population BP (34). Substantial evidence shows that sodium reduction lowers BP and controls hypertension (13). Likewise, a high-potassium diet, such as the DASH diet (35), reduces BP and can help to control hypertension. In addition to lowering BP, a high potassium intake blunts the effects of a high sodium intake on BP (39). For individuals already receiving antihypertensive treatment, a low-salt diet may reduce the dosage of antihypertensive medications required to maintain BP control (13). In this context, there is a need for effective public health initiatives to promote and achieve diets reduced in sodium and rich in potassium as a means to lower BP and prevent its cardiovascular and renal consequences.

Our study results indicate that the consumption of potassium-rich foods is insufficient in Bangladesh. According to the national WHO-STEPS survey (2018), the average daily intake of fruits and vegetables among adults was only 2.6 servings, and the prevalence of an adequate intake of fruits and vegetables per day (i.e., at least five servings a day) was extremely low at 10.4% (36). Available evidence indicates that increasing potassium intake can lower BP among those with hypertension (37,38). The most significant reduction in BP is observed when daily potassium intake increases by about 90–120 mmol (90 mmol = 3.5g) (15). Nevertheless, the strong positive link between BP and cardiovascular disease, as well as between BP and coronary heart disease, indirectly suggests that increasing potassium intake can potentially prevent these outcomes by lowering BP (15).

Potassium-enriched salt substitutes have the potential to decrease the amount of dietary sodium people consume while increasing their potassium intake. In many countries, particularly those in South and Southeast Asia, potassium-rich salt substitutes are being used instead of regular sodium chloride to reduce discretionary salt at the household level (39,40). Similarly, many food manufacturers should be able to substitute potassium-enriched salt for traditional salt, thereby lowering sodium intake and increasing potassium intake in processed foods (40). Implementation of population-wide potassium-enriched salt substitute intervention can significantly reduce hypertension and prevent cardiovascular deaths (41). The approach to integrating potassium-enriched salt substitutes into public health initiatives will vary from one country to another, depending on the primary sources of sodium in people’s diets (40).

Salt reduction interventions are deemed to be cost-effective, particularly in South and Southeast Asia (27). Targeted community-based salt reduction programs tailored to the local context can be implemented. Health education and awareness campaigns should highlight the effects of excess salt consumption on health. In South Asia, adding salt during cooking and at the table before eating is a common dietary practice (27). In Bangladesh, most people habitually add table salt to their cooked meals (42). In Bangladesh, 80% of salt was used during cooking or at the table (27,42). These findings highlight the critical need for public health interventions to address and mitigate excessive salt consumption in the country.

Policy changes and associated education campaigns following successful models from various countries are needed to bring changes in dietary behavior (27). Bangladesh has taken proactive steps towards salt reduction by initiating multi-sectoral plans. These include food reformulation, discouraging the sale of high-salt processed foods, and launching population education through mass media campaigns (35). Furthermore, given the success of school-based health education programs integrated into China’s regular curriculum (43), the Bangladesh government might consider adopting similar strategies to promote behavior change. In addition, one effective policy tool that governments can use to regulate products and help people maintain a balanced diet is the implementation of front-of-package warning labels (44). This labeling system informs consumers about the levels of salt or sodium, sugar, and other nutrients present in specific products (44).

The study’s strengths include representation from both urban and rural areas across several regions of the country and the use of 24-hr urine collection—the gold-standard method to estimate dietary salt and potassium intake (17). Previous studies in Bangladesh have estimated dietary sodium and salt intake using the 24-hr urinary excretion method (24,27). Potassium intake has previously been estimated using the Tanaka spot urine method (45). This is the first study to assess dietary potassium intake from 24-hr urinary excretion among Bangladeshi adults.

Limitations of our study include the collection of a single 24-hr urine sample, which cannot estimate usual sodium and potassium intakes at the individual level due to high intra-individual variability in electrolyte excretion (35). However, a single measurement is sufficient to estimate sodium and potassium intake at the population level. Second, while the study design and sample size are appropriate for estimating population-level intake of sodium and potassium, the study was underpowered to detect associations of sodium and potassium intake with BP and hypertension. Third, while 100% of dietary sodium is excreted in the urine, the fraction of dietary potassium excreted in the urine varies considerably and is estimated to be 70% on average (19,46). Hence, estimated dietary potassium intake is less precise than estimated dietary sodium (and salt) intake. Fourth, the final sample size was smaller than initially planned.

## Conclusions

In our study, mean salt intake was almost twice the upper limit recommended by WHO, while mean potassium intake was substantially below the minimum dietary recommendation. These findings provide a strong rationale for launching public health initiatives that simultaneously reduce sodium and increase potassium intake to lower BP and prevent hypertension and, ultimately, their downstream CVD consequences in Bangladesh. Such initiatives could include the promotion of potassium-enriched salt substitutes as a substitute for traditional salt.
